# Pavlovian threat learning shapes the kinematics of action

**DOI:** 10.3389/fpsyg.2022.1005656

**Published:** 2022-10-11

**Authors:** Francesca Starita, Sara Garofalo, Daniela Dalbagno, Luigi A. E. Degni, Giuseppe di Pellegrino

**Affiliations:** Motivation, Decision and Learning Laboratory, Center for Studies and Research in Cognitive Neuroscience, Department of Psychology “Renzo Canestrari,” University of Bologna, Cesena, Italy

**Keywords:** Pavlovian conditioning, fear conditioning, kinematics, reaching, vigor, pain anticipation

## Abstract

Prompt response to environmental threats is critical to survival. Previous research has revealed mechanisms underlying threat-conditioned physiological responses, but little is known about how threats shape action. Here we tested if threat learning shapes the kinematics of reaching in human adults. In two different experiments conducted on independent samples of participants, after Pavlovian threat learning, in which a stimulus anticipated the delivery of an aversive shock, whereas another did not, the peak velocity and acceleration of reaching increased for the shocked-paired stimulus, relative to the unpaired one. These kinematic changes appeared as a direct consequence of learning, emerging even in absence of an actual threat to body integrity, as no shock occurred during reaching. Additionally, they correlated with the strength of sympathetic response during threat learning, establishing a direct relationship between previous learning and subsequent changes in action. The increase in velocity and acceleration of action following threat learning may be adaptive to facilitate the implementation of defensive responses. Enhanced action invigoration may be maladaptive, however, when defensive responses are inappropriately enacted in safe contexts, as exemplified in a number of anxiety-related disorders.

## Introduction

“Diede un’occhiata, al di sopra del muricciolo, ne’ campi: nessuno; un’altra più modesta sulla strada dinanzi; nessuno, fuorché i bravi. Che fare? tornare indietro, non era a tempo: darla a gambe, era lo stesso che dire, inseguitemi, o peggio. Non potendo schivare il pericolo, vi corse incontro, perché i momenti di quell’incertezza erano allora così penosi per lui, che non desiderava altro che d’abbreviarli.” I Promessi Sposi, Alessandro Manzoni

“He cast a glance over the low wall into the fields — no one; another, more subdued, along the path forward — no one but the bravoes. What is to be done? turn back? It is too late. Run? It was the same as to say, follow me, or worse. Since he could not escape the danger, he went to meet it. These moments of uncertainty were already so painful, he desired only to shorten them.” The Betrothed, Alessandro Manzoni

Environmental stimuli may acquire threat-related properties and exert a powerful influence on behavior. For example, after facing our neighbor’s dog growling at us, we may walk faster than usual when passing in front of its house, fearing a new encounter with the vicious animal. In the laboratory, Pavlovian threat learning (fear conditioning) is a well-validated procedure through which intrinsically neutral stimuli acquire threat value through pairing with an aversive event, eliciting changes in physiological response, subjective experience, and overt behavior (Pavlov, 1927; Maren, 2001; Lonsdorf et al., 2017). Notably, while extensive research has investigated the changes in physiological response and subjective experience, the study of changes in overt behavior has largely been neglected in humans (Beckers et al., 2013; Lonsdorf et al., 2017).

In fact, Pavlovian threat learning is known to elicit innate, automatic defensive motor reflexes and reactions (Blumenthal et al., 2005; Zhang et al., 2016; Lonsdorf et al., 2017; Roelofs, 2017), as the presence of a stimulus signaling imminent danger can trigger active defensive motor responses, such as fight or flight, mediated by an increase in arousal and a burst in sympathetic activity (Fanselow, 1994; Rau and Fanselow, 2007; Reker et al., 2010; Garofalo et al., 2015, 2017; Magosso et al., 2015; LeDoux and Daw, 2018; Hamm, 2020; Mobbs et al., 2020). However, in humans, the investigation of such responses has been mostly conducted by means of simple reaction time tasks (Krypotos et al., 2014, 2015a; Pittig et al., 2020), which limit the investigation to pre-movement processes. Indeed, motor processes include a pre-movement phase, during which an action is selected and its execution planned, but also a separate movement phase, in which the action is executed, and which is described by its kinematics (Elliott et al., 2010; Rosenbaum, 2010; Wong et al., 2015). Notably, although execution emerges from planning, the two processes can be independently affected as reflected by dissociable changes in reaction times and kinematics (e.g., Betti et al., 2018). Thus, in order to advance the understanding of the mechanisms underlying adaptive and maladaptive learning, the investigation of action kinematic may provide useful to test whether and how Pavlovian threat learning affects motor control beyond reaction times and action planning.

On this basis, the main aim of the present study is to investigate the effect of Pavlovian acquired threat value on the kinematics, of the reaching movement, one of the most common goal-directed actions performed in daily life. Importantly, action kinematic is modulated by the action’s goal (Ansuini et al., 2006; Sartori et al., 2011), and, in addition to the physical features of the goal (e.g., shape, size, and position of the target object) (Jakobson and Goodale, 1991; Castiello, 2005), its motivational value also shapes action kinematic (Ferri et al., 2010; Esteves et al., 2016; Karos et al., 2017; Nishi et al., 2021). In particular, regarding threat value, acting toward an aversive stimulus has been shown to affect both the planning and execution of the movement, such that movement reaction time, velocity, acceleration, deceleration, and accuracy have been found to increase when moving in a direction associated with the delivery of an aversive somatosensory stimulation (Karos et al., 2017; Neige et al., 2018; but see Nishi et al., 2021). Such effect has been explained in terms of a “get it over and done with” motor strategy in order to minimize the duration of the pain-associated response, given the fact that the aversive stimulation could not be avoided or escaped (Karos et al., 2017; Neige et al., 2018).

Here, we exploit the methods used in the literature on motor control to test whether similar changes in kinematics occur even when the goal of the action is not intrinsically aversive, but instead is a conditioned stimulus (CS), i.e., an intrinsically neutral stimulus that has acquired threat value through pairing with an aversive event (e.g., shock) during Pavlovian learning. Additionally, we investigate the extent to which these changes are a direct consequence of learning, appearing to the mere presence of the CS, or whether they appear only when in presence of an actual threat from the environment. In this regard, reaction time tasks (Krypotos et al., 2014, 2015a) seem to suggest that Pavlovian acquired threat value may automatically and inflexibly shape motor response, even in absence of any environmental threat. Two experiments were conducted.

## Experiment 1

Here, participants first completed a baseline phase, in which they reached with the computer mouse two different neutral stimuli on the screen. A Pavlovian threat learning phase followed, during which participants learned to identify one stimulus as threatening (CS+), as it was paired with an aversive shock, while the other stimulus was never paired with shock (CS−). Note that no voluntary movement was performed during this phase, and thus action performance and aversive stimulation are never experienced together. Finally, during two test phases, participants reached the two stimuli again. To test the extent to which kinematic changes are a direct consequence of learning, in one test phase, reaching was performed under safety (i.e., without the shock electrodes attached to the wrist; Gillan et al., 2014; Krypotos et al., 2014, 2015a; Garofalo and Robbins, 2017), while in the other, reaching was performed under threat of shock delivery (i.e., with the shock electrodes attached to their wrist). Note that no shock was ever delivered in either block, and thus action performance is tested in absence of any aversive stimulation.

During baseline and tests phases, reaching trajectory and timing was recorded in order to compute reaction time, peak velocity, acceleration, deceleration, and end-point accuracy. During the Pavlovian threat learning phase, skin conductance response (SCR), a measure of sympathetic response and arousal (Critchley et al., 2000), was recorded in order to assess successful learning (i.e., greater SCR to CS+ than CS−). In line with the results of the studies described above (Starita et al., 2016; Karos et al., 2017; Neige et al., 2018), we hypothesized an increase in reaction time, peak velocity, acceleration, deceleration, and greater accuracy when reaching the CS+ than the CS−. Additionally, if the kinematic changes are sensitive to the presence of actual threat, they should be evident at test under threat of shock delivery but not under safety.

### Materials and methods

#### Participants

Thirty-four healthy participants (17 males; age *M* = 25.11 years, SD = 4.17 years, all right-handed assessed through self-report) were randomly selected from the local population to complete the study, based on previous studies investigating the relationship between aversive stimuli or threat learning and motor response (Krypotos et al., 2014; Karos et al., 2017; Nishi et al., 2021). The study followed the American Psychological Association Ethical Principles of Psychologists and Code of Conduct and the Declaration of Helsinki and was approved by the Bioethics Committee of the University of Bologna (protocol number 224364). All participants provided written informed consent to participation.

##### Stimuli

The stimuli appeared on a 43-inches computer screen (resolution: 1,920 × 1,080; refresh rate: 60 Hz), at a viewing distance of ∼60 cm. A PC running OpenSesame software (Mathôt et al., 2012) controlled the flow of the task. In all phases, stimuli were a yellow or a pink circle (64 pixels diameter, equivalent to 3.17 cm) appearing on each trial in one out of three possible locations on the screen (low, middle, and high).

#### Experimental task

**FIGURE 1:**
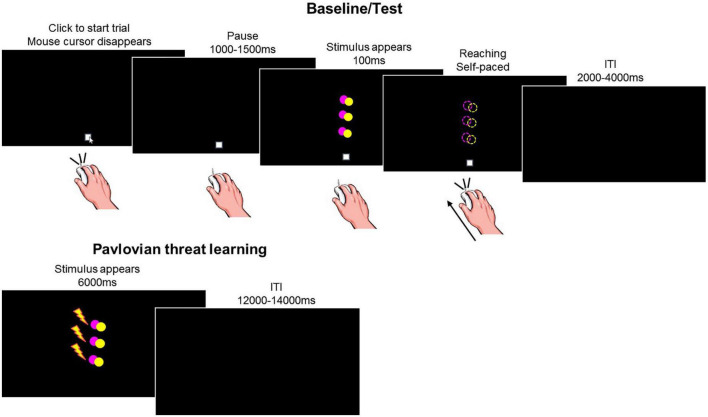
Experimental task. During baseline and test, participants made reaching movements with the computer mouse toward the stimuli appearing on the screen. The task included two colored circles (yellow or pink). On each trial, one of the circles appeared in one out of three positions (low, middle, and high) for 100 ms. We show an example of a stimulus in the low, middle and high positions, represented together on the same screen for illustrative purposes only. During Pavlovian threat learning, participants learned to associate a specific stimulus color with an aversive outcome (i.e., shock, represented by the lightning), while the other color served as within-subject control condition. The task included two colored circles (yellow or pink). On each trial, one of the circles appeared in one out of three positions (low, middle, and high). We show an example of a stimulus in the low, middle, and high positions, represented together on the same screen for illustrative purposes only. Dashed circles and the lightning are for illustrative purposes only, they were not present during the actual task.

The task included four consecutive phases: baseline, Pavlovian threat learning, test under safety, test under threat (Figure 1).

##### Baseline phase

Participants completed a computerized task in which they used a mouse to reach (from a starting point) visual stimuli appearing on the screen. On each trial, the start position appeared (*xy* center coordinates 0, −416 pixels) with the mouse cursor on it. Participants then clicked on the start position and, after a jittered interval of 1–1.5 s, a yellow or pink circle appeared for 100 ms centered at one of three possible positions (low 0, −96 *xy* pixels, middle 0, 64 *xy* pixels, high 0, 224 *xy* pixels) and participants reached the stimulus. A black screen ended the trial (jittered 2–4 s intertrial interval). Note that as soon as participants made the click on the starting position, the icon of the mouse cursor disappeared from the screen. Thus, reaching was performed in absence of visual feedback regarding cursor position, to reduce the possibility of movement correction after its initiation (Elliott et al., 2010). Additionally three target positions where included to reduce the possibility that the movement is programmed before the presentation of the visual stimulus (Cisek and Kalaska, 2005).

The following instructions were given at the beginning of the phase: “You will see a colored circle briefly appearing on the screen. Your task is to reach with the mouse the position where the circle appeared and click on it. You will have to make a single, continuous movement and be as fast and accurate as possible.”

This phase included 20 trials per condition (total 120 trials) presented in random order. The Mousetrap plugin (Kieslich and Henninger, 2017) for OpenSesame was used to track the mouse cursor’s position over time, at a frequency of 100 Hz (default option). Note that on each click on the starting position, the mouse was centered on 0, −416 coordinate, to ensure a common starting point among trials.

##### Pavlovian threat learning phase

Participants learned to identify a specific stimulus color as threatening, i.e., conditioned stimulus (CS+), while the other color served as within-subject control condition (CS−). Color assignment to each CS role was counterbalanced between participants. Presentation of the CS+ co-terminated with the delivery of the US in 7 out of 9 trials (77.8% reinforcement rate). Presentation of the CS− was never paired with the US.

The unconditioned stimulus (US) consisted of a 2 ms aversive electrical shock generated by a Digitimer Stimulator (Model DS7A, Digitimer Ltd., UK) and delivered to the participants’ left wrist through pre-gelled Ag/AgCl snapped electrodes (Friendship Medical, SEAg-S-15000/15 × 20). The US intensity (*M* = 35.02 mA, SD = 17.70) was calibrated for each participant to a level deemed “highly unpleasant, but not painful” using an ascending staircase procedure. Participants rated the unpleasantness of the shock (*M* = 7.68, SD = 0.73) on a scale ranging from 0 (*no sensation*) to 10 (*painful*).

Participants were then instructed that the circles would appear one at the time on the screen and might be associated with the shock, and that their task was to pay attention to the screen and try to predict which circle would give them the shock. Note that no information was provided regarding which color would be associated with the shock, and participants had to learn the CSs-US relationship from experience. Also, participants made no motor responses but only observed what happened on the screen.

This phase included 9 trials per condition (total 54 trials) and each CS was presented for 6 s followed by a jittered 12–14 s inter-trial interval. Except for the first six acquisition trials that started with three CS− trials and three reinforced CS+ trials (one for each position), in random order, trials proceeded in pseudo-random order, such that no more than two consecutive stimuli of the same type occurred in a row. Skin conductance, subjective reports of CS valence, and CS-US contingency awareness were recorded (see dependent measures).

##### Test phases under safety and threat

There were two test phases, which were exactly as the baseline phase, except that one phase was completed under safety (i.e., without the shock electrodes), while the other was under the threat of shock delivery (i.e., with the shock electrodes attached to the wrist of the left hand). Note that during the test phase under threat of shock, no shock was ever delivered. The order of the two test phases was counterbalanced among participants.

#### Procedure

Participants were comfortably seated in a silent and dimly lit room, and their position was centered relative to the computer screen. Participants completed a practice before starting the task to familiarize themselves with the reaching movements (total 18 trials). The practice was structured as the baseline phase except that the circles were white and that the mouse cursor was visible for the entire duration of the trial. Then, participants completed the baseline phase. Once completed, electrodes for SCR recording and shock delivery were attached to them. After verifying the correct recording of SCR, the shock intensity was calibrated. The Pavlovian threat learning phase followed. The test followed, and shock electrodes were removed in case of the test under safety, while they were left on participants’ wrist or reattached (if test under safety came first) in case of the test under threat (order of the two test phases counterbalanced among participants). Finally, participants completed subjective ratings of CS valence, and awareness of the CS-US contingencies (see dependent variables).

#### Dependent variables

##### Pavlovian threat learning

###### Skin conductance response

Galvanic skin conductance was recorded during the entire phase at 1,250 Hz, with a 10 Hz low-pass filter, from pre-gelled snap electrodes (BIOPAC EL501) placed on the hypothenar eminence of the palmar surface of the non-shocked hand, connected to a BIOPAC MP-150 System (Goleta, CA, USA). The digitalized signal was down-sampled at 200 Hz and processed using Autonomate 2.8 (Green et al., 2014) to obtain trough-to-peak SCR values. A SCR was considered valid if the trough-to-peak deflection started between 0.5–4.5 s following the CS onset, lasted for a maximum of 5 s, and was greater than 0.02 μS. Trials that did not meet these criteria were scored as zero (Starita et al., 2019). Data from one subject were not recorded due to PC malfunctioning.

###### Explicit conditioned stimulus-unconditioned stimulus contingency awareness

To evaluate explicit learning of the CS-US contingency, at the end of the phase, participants saw each CS, and made a forced-choice response (yes/no) to the question “when the circle was of this color it gave me the shock.” All participants except one gave the correct response. In particular, that participant responded “yes” also to the CS−; importantly, its inclusion in the analysis did not affect the results’ significance.

###### Subjective ratings of conditioned stimuli valence

To have an explicit measure of the subjective experience of CSs, at the end of the phase, participants saw each CS and answered the question “the feeling I had when the circle was of this color was,” on an 11 point Likert scale ranging from −5 (unpleasant) to +5 (pleasant), with 0 representing neutral.

#### Kinematics

Participants’ raw 2-D mouse-tracking data were extracted from the OpenSesame logger using the mousetrap package (https://github.com/pascalkieslich/mousetrap/) in RStudio and produce a derivative.csv file, with the timestamp, *x* and *y* coordinates, velocity, and acceleration across both *x* and *y* dimensions of each sampled point. These data were then imported in MATLAB and processed using custom-made scripts. First, the trajectory and velocity profile of each trial was visually inspected to exclude trials showing abnormal responses, such as erratic movements atypical for this context, e.g., with multiple left/right or up/down deviations from a straight line (Kieslich et al., 2018). Because participants had to perform a mouse click in order to proceed with the task, there were no missed responses. Visual inspection of anomalous trials led to the exclusion of *M* = 1.38 trials (min 0, max 12) for the baseline phase, *M* = 0.88 trials (min 0, max 7) for the test without electrodes phase and *M* = 1.03 trials (min 0, max 6 per participant) for the test with electrodes phase. Then, from each of the remaining trials we extracted the following parameters: peak velocity, peak acceleration, peak deceleration, reaction time (defined as the time from stimulus offset to the first point in time where velocity was equal to or exceeded 2% of its peak; Ferri et al., 2010), and movement accuracy (defined as the Euclidean distance between the center of the stimulus and the position of the mouse where velocity was equal to or fell below 2% of its peak). Finally, trials were separated between the six experimental conditions (i.e., 3 positions, 2 types of CS) and averaged for each participant.

#### Statistical analyses

Analyses were performed with JASP 0.14.1.0 (JASP Team, 2020). Data distributions were visually inspected for normality. Repeated-measures analyses of variance (RM ANOVA) were used to investigate differences between more than two conditions followed by planned contrasts, wherever appropriate, while paired *t*-tests were used to investigate differences between only two conditions. Degrees-of-freedom and *p*-values were Greenhouse–Geisser corrected, whenever a violation of the sphericity assumption occurred. Partial eta-squared ($\eta_{p}^{2}$) and 90% confidence intervals (CI) were computed as estimates of effect sizes for the ANOVAs’ main effects and interactions, while Cohen’s *d* and 95% CI for the *t*-tests (Lakens, 2013). A statistical significance threshold of *p* < 0.05 was adopted.

### Results

#### Kinematics at baseline

**FIGURE 2:**
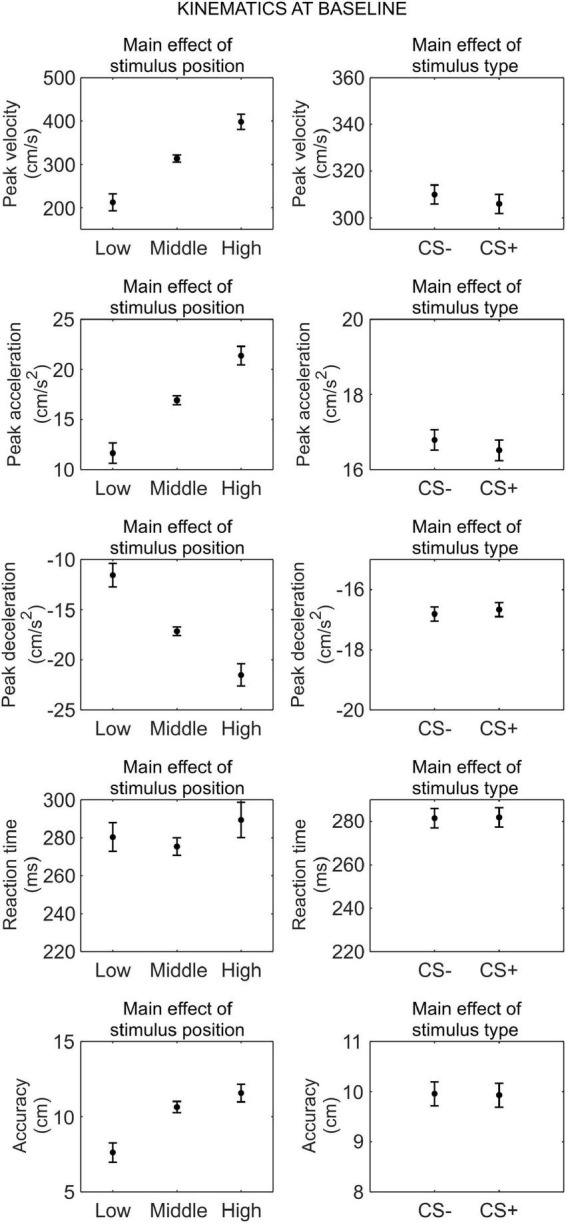
Kinematics at baseline. Each plot shows the group mean (black dot) and 95% confidence intervals (vertical bars) of the different kinematic parameters as a function of the stimulus position or the stimulus type. 95% CI were corrected for within-subjects designs (Cousineau, 2005).

We conducted a series of 3 (stimulus position: low, middle, high) × 2 (stimulus type: CS+, CS−) RM ANOVA to assess whether there was any difference in kinematics between the CS+ and CS−, before threat learning (Figure 2).

For peak velocity, we observed a main effect of position (*F*_(1_._26_,_41_._72)_ = 83.52, *p* < 0.001, $\eta_{p}^{2}=0.72$, 90% CI [0.58, 0.78]). Helmert contrasts showed that participants were slower with the low position than the middle and high positions (*t*_(66)_ = −11.51, *p* < 0.001), and for the middle position than the high position (*t*_(66)_ = −5.88, *p* < 0.001; *M*_*low*_ = 212.33 cm/s, SD*_*low*_* = 122.49 cm/s; *M*_*middle*_ = 313.44 cm/s, SD*_*middle*_* = 174.83 cm/s; *M*_*high*_ = 398.04 cm/s, SD*_*high*_* = 208.72 cm/s). No other main effect or interaction was significant (all *p* ≥ 0.354).

For peak acceleration, we observed a main effect of position (*F*_(1_._30_,_42_._97)_ = 84.77, *p* < 0.001, $\eta_{p}^{2}=0.72$, 90% CI [0.58, 0.79]). Helmert contrasts showed that participants accelerated less with the low position than the middle and high positions (*t*_(66)_ = −11.58, *p* < 0.001), and for the middle position than the high position (*t*_(66)_ = −5.96, *p* < 0.001; *M*_*low*_ = 11.65 cm/s^2^, SD*_*low*_* = 6.58 cm/s^2^; *M*_*middle*_ = 16.93 cm/s^2^, SD*_*middle*_* = 9.34 cm/s^2^; *M*_*high*_ = 21.38 cm/s^2^, SD*_*high*_* = 11.17 cm/s^2^). No other main effect or interaction was significant (all *p* ≥ 0.346).

For peak deceleration, we observed a main effect of position (*F*_(1_._21_,_39_._90)_ = 65.75, *p* < 0.001, $\eta_{p}^{2}=0.67$, 90% CI [0.50, 0.75]). Helmert contrasts showed that participants decelerated more for the low position than the middle and high positions (*t*_(66)_ = 10.32, *p* < 0.001), and for the middle position than the high position (*t*_(66)_ = 5.00, *p* < 0.001; *M*_*low*_ = −11.55 cm/s^2^, SD*_*low*_* = 6.76 cm/s^2^; *M*_*middle*_ = −17.15 cm/s^2^, SD*_*middle*_* = 10.25 cm/s^2^; *M*_*high*_ = −21.51 cm/s^2^, SD*_*high*_* = 12.45 cm/s^2^). No other main effect or interaction was significant (all *p* ≥ 0.544).

For reaction time, we observed no significant main effect or interaction (all *p* ≥ 0.100).

For accuracy, we observed a main effect of position (*F*_(1_._54_,_50_._84)_ = 35.47, *p* < 0.001, $\eta_{p}^{2}=0.52$, 90% CI [0.34, 0.62]). Helmert contrasts showed greater accuracy for the low position than the middle and high positions (*t*_(66)_ = −8.21, *p* < 0.001), and no significant difference between the middle and high positions (*t*_(66)_ = −1.89, *p* = 0.063; *M*_*low*_ = 7.62 cm, SD*_*low*_* = 6.94 cm; *M*_*middle*_ = 10.64 cm, SD*_*middle*_* = 7.68 cm; *M*_*high*_ = 11.57 cm, SD*_*high*_* = 6.79 cm). No other main effect or interaction was significant (all *p* ≥ 0.227).

Taken together, these results indicate that, before threat learning, there was no significant difference in kinematic between CS+ and CS−.

#### Pavlovian threat learning

**FIGURE 3:**
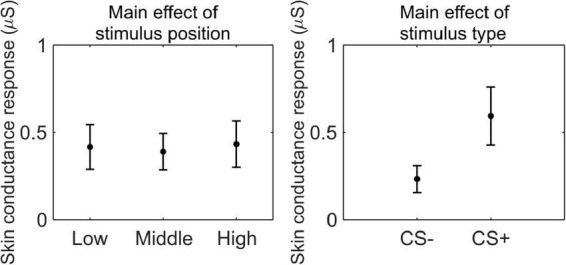
Skin conductance response during threat learning. Each plot shows the group mean (black dot) and 95% confidence intervals (vertical bars) of the skin conductance response as a function of the stimulus position or the stimulus type.

We conducted a 3 (stimulus position: low, middle, high) × 2 (stimulus type: CS+, CS−) RM ANOVA to test whether SCR was modulated by CSs association (or absence thereof) with the US, as a function of its position. We observed a main effect of stimulus type (*F*_(1_,_33)_ = 41.41, *p* < 0.001, $\eta_{p}^{2}=0.56$, 90% CI [0.35, 0.67]), indicating higher SCR for CS+ than CS− (CS+: *M* = 0.59 μS, SD = 0.50 μS; CS−: *M* = 0.23 μS, SD = 0.23 μS; Figure 3).

We conducted a paired *t*-test to test whether subjective ratings of stimulus valence were modulated by CSs association (or absence thereof) with the US. Participants rated the CS+ (*M* = −2.76, SD = 1.60) as less pleasant than the CS− (*M* = 1.21, SD = 1.70; *t*_(33)_ = 8.53, *p* < 0.001, *d* = 1.46, 95% CI [0.97, 1.94]).

These results indicate that participants successfully acquired threat learning, showing higher arousal to the CS+ than the CS− and rating the CS+ as less pleasant than CS−.

#### Kinematics at test

**FIGURE 4:**
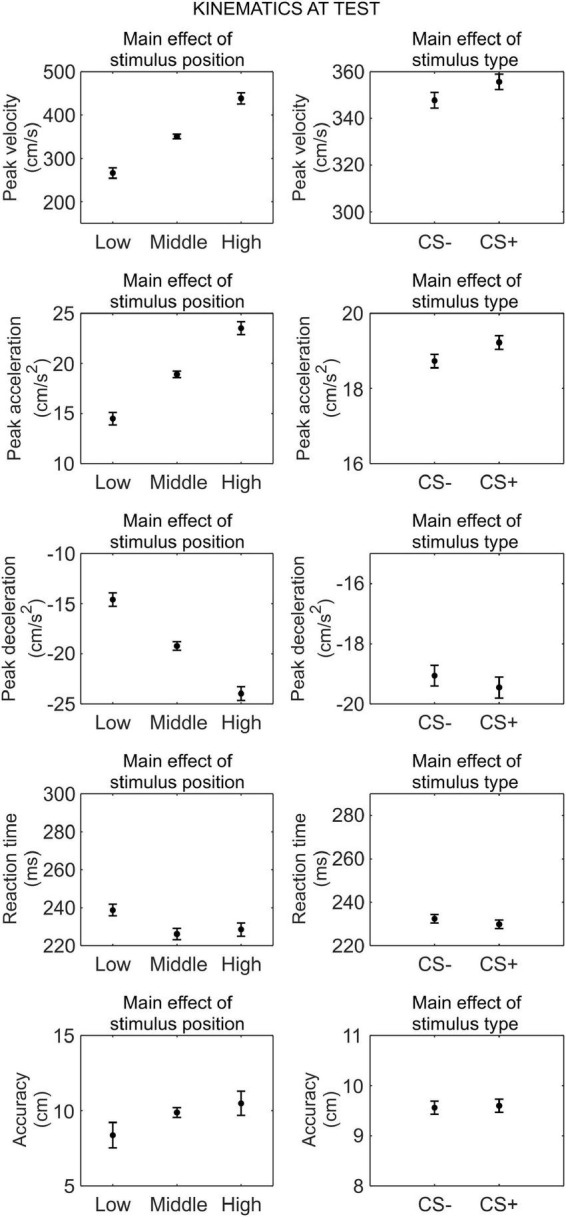
Kinematics at test. Each plot shows the group mean (black dot) and 95% confidence intervals (vertical bars) of the different kinematic parameters as a function of the stimulus position or the stimulus type. 95% CI were corrected for within-subjects designs (Cousineau, 2005).

We conducted a series of 2 (phase: test under safety, test under threat) × 3 (stimulus position: low, middle, high) × 2 (stimulus type: CS+, CS−) RM ANOVA to assess differences in kinematics between the CS+ and CS−, after threat learning (Figure 4).

For peak velocity, we observed a main effect of position (*F*_(1_._30_,_42_._74)_ = 164.09, *p* < 0.001, $\eta_{p}^{2}=0.83$, 90% CI [0.75, 0.87]). Helmert contrasts showed that participants were slower with the low position than the middle and high positions (*t*_(66)_ = −15.59, *p* < 0.001), and with the middle position than the high position (*t*_(66)_ = −9.22, *p* < 0.001; *M*_*low*_ = 266.16 cm/s, SD*_*low*_* = 220.27 cm/s; *M*_*middle*_ = 350.66 cm/s, SD*_*middle*_* = 235.68 cm/s; *M*_*high*_ = 438.27 cm/s, SD*_*high*_* = 238.16 cm/s). Crucially, we also observed a main effect of stimulus type (*F*_(1_,_33)_ = 5.22, *p* = 0.029, $\eta_{p}^{2}=0.14$, 90% CI [0.01, 0.31]), showing that participants were faster with the CS+ (*M* = 355.65 cm/s, SD = 233.13 cm/s) than the CS− (*M* = 347.74 cm/s, SD = 225.83 cm/s), regardless of the test phase. No other main effect or interaction was significant (all *p* ≥ 0.121).

For peak acceleration, we observed a main effect of position (*F*_(1_._40_,_46_._09)_ = 167.95, *p* < 0.001, $\eta_{p}^{2}=0.84$, 90% CI [0.75, 0.87]). Helmert contrasts showed that participants accelerated less with the low position than the middle and high positions (*t*_(66)_ = −15.72, *p* < 0.001), and with the middle position than the high position (*t*_(66)_ = −9.31, *p* < 0.001; *M*_*low*_ = 14.49 cm/s^2^, SD*_*low*_* = 11.88 cm/s^2^; *M*_*middle*_ = 18.92 cm/s^2^, SD*_*middle*_* = 12.90 cm/s^2^; *M*_*high*_ = 23.52 cm/s^2^, SD*_*high*_* = 12.97 cm/s^2^). Crucially, we also observed a main effect of stimulus type (*F*_(1_,_33)_ = 7.07, *p* = 0.012, $\eta_{p}^{2}=0.18$, 90% CI [0.02, 0.35]), showing that participants accelerated more with the CS+ (*M* = 19.22 cm/s^2^, SD = 12.73 cm/s^2^) than the CS− (*M* = 18.73 cm/s^2^, SD = 12.25 cm/s^2^), regardless of the test phase. No other main effect or interaction was significant (all *p* ≥ 0.079).

For peak deceleration, we observed a main effect of position (*F*_(1_._61_,_53_._08)_ = 148.06, *p* < 0.001, $\eta_{p}^{2}=0.82$, 90% CI [0.73, 0.86]). Helmert contrasts showed that participants decelerated less with the low position than the middle and high positions (*t*_(66)_ = 14.83, *p* < 0.001), and with the middle position than the high position (*t*_(66)_ = 8.73, *p* < 0.001; *M*_*low*_ = −14.59 cm/s^2^, SD*_*low*_* = 12.47 cm/s^2^; *M*_*middle*_ = −19.21 cm/s^2^, SD*_*middle*_* = 13.63 cm/s^2^; *M*_*high*_ = −23.964 cm/s^2^, SD*_*high*_* = 14.48 cm/s^2^). No other main effect or interaction was significant (all *p* ≥ 0.132).

For reaction time, we observed a main effect of position (*F*_(2_,_66)_ = 11.35, *p* < 0.001, $\eta_{p}^{2}=0.26$, 90% CI [0.10, 0.38]). Helmert contrasts showed longer reaction time for the low position than the middle and high positions (*t*_(66)_ = 4.69, *p* < 0.001), and no significant difference between the middle and high positions (*t*_(66)_ = −0.83, *p* = 0.408; *M*_*low*_ = 238.77 ms, SD*_*low*_* = 63.16 ms; *M*_*middle*_ = 226.12 ms, SD*_*middle*_* = 57.95 ms; *M*_*high*_ = 228.47 ms, SD*_*high*_* = 66.05 ms). No other main effect or interaction was significant (all *p* ≥ 0.135).

For accuracy, we observed a main effect of position (*F*_(1_._24_,_40_._81)_ = 6.03, *p* = 0.013, $\eta_{p}^{2}=0.15$, 90% CI [0.02, 0.31]). Helmert contrasts showed greater accuracy for the low position than the middle and high positions (*t*_(66)_ = −3.33, *p* = 0.001), and no significant difference between the middle and high positions (*t*_(66)_ = −0.98, *p* = 0.332; *M*_*low*_ = 8.37 cm, SD*_*low*_* = 9.69 cm; *M*_*middle*_ = 9.88 cm, SD*_*middle*_* = 8.21 cm; *M*_*high*_ = 10.49 cm, SD*_*high*_* = 7.42 cm). No other main effect or interaction was significant (all *p* ≥ 0.519).

Taken together, these results indicate that, after threat learning, reaching kinematic was still modulated according to the position of the stimulus, and, crucially, participants moved faster and accelerated more when reaching the CS+ than the CS−. In contrast, no difference between CS+ and CS− was found for deceleration, reaction time, and accuracy.

### Summary of Experiment 1

The aim of Experiment 1 was to test whether Pavlovian threat learning shapes the kinematics of goal-directed actions. The first result was that reaching kinematic was modulated according to the position of the stimulus, confirming compliance with the task, showing an increase in peak velocity, acceleration and deceleration, from closer to farther stimuli, in line with reaching in real-life (Jakobson and Goodale, 1991), suggesting a resemblance between mouse tracking and real-life data. Importantly, we found that following learning, peak velocity and acceleration increased when reaching the conditioned stimulus (CS+) as compared to the control stimulus (CS−). This invigoration of reaching occurred even though action performance did not *per se* lead to an aversive consequence or the avoidance of such consequence, as the shock was never delivered at test. We also found no evidence that the invigoration was sensitive to the presence of an actual threat, as it was observed regardless of the test (threatening vs. safe test) in which the action was performed. The mere presence of the conditioned stimulus shaped the action, even in absence of a real threat to body integrity.

## Experiment 2

We then wondered the extent to which the invigoration observed in Experiment 1 depended on the presence of the baseline. During the baseline, participants reach the stimuli, creating a stimulus-response association. Given this, it may be possible that, during the Pavlovian learning phase, participants do not simply create a stimulus-outcome association, but also a stimulus-response-outcome association. Thus, in Experiment 2, we tested whether the existence of a stimulus-response association before threat learning plays a causal role in the emergence of the invigoration. To this end, a different group of participants performed the same task as Experiment 1, but without the baseline phase. Replication of the invigoration when reaching the CS+ will suggest that the baseline phase plays no causal role in determining the kinematic changes and that a stimulus-response association is not necessary to observe them.

### Materials and methods

#### Participants

A different independent sample of thirty-four healthy participants (14 males; age *M* = 22.81 years, SD = 3.08 years, all right-handed assessed through self-report) completed the study. The study followed the American Psychological Association Ethical Principles of Psychologists and Code of Conduct and the Declaration of Helsinki and was approved by the Bioethics Committee of the University of Bologna (protocol number 224364). All participants provided written informed consent to participation.

#### Experimental task

The task was exactly as Experiment 1, except that there was no baseline phase. Thus, participants completed the Pavlovian threat learning phase, followed by the two test phases, under the threat of shock (i.e., with the shock electrodes attached to the wrist of the left hand) and under safety (i.e., without the shock electrodes; order counterbalanced among participants). As in Experiment 1, no shock was ever delivered during the test phases. For the Pavlovian threat learning phase, the US intensity was *M* = 32.98 mA, SD = 14.15 and the unpleasantness of the shock was rated *M* = 7.97, SD = 0.67. Also, all participants reported the correct CS-US contingencies.

#### Dependent variables

##### Pavlovian threat learning

We collected all the variables included in Experiment 1 and added a subjective rating of shock expectancy at test as described below.

##### Subjective ratings of shock expectancy at test with electrodes

To have an explicit measure of the expectancy of receiving a shock during the test phase with the electrodes attached to the wrist, participants saw each stimulus and answered the following question at the end of the task, “how much did you expect the shock when you moved the mouse with the electrodes attached to the wrist and the circle was of this color,” on an 11 point Likert scale ranging from −5 (not at all) to +5 (a lot), with 0 representing neutral.

#### Kinematics

Data processing was exactly as Experiment 1. Visual inspection of anomalous trials led to the exclusion of *M* = 2.97 trials (min 0, max 11 per participant) for the test without electrodes phase and *M* = 2.91 trials (min 0, max 7 per participant) for the test with electrodes phase.

#### Statistical analyses

They were the same as Experiment 1.

### Results

#### Pavlovian threat learning

**FIGURE 5:**
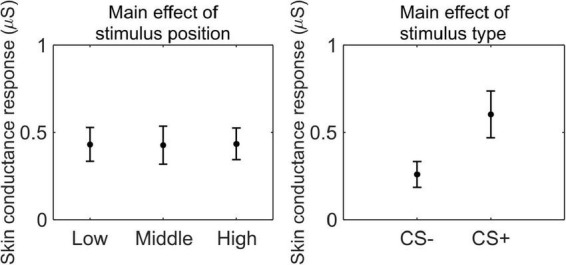
Skin conductance response during threat learning. Each plot shows the group mean (black dot) and 95% confidence intervals (vertical bars) of the skin conductance response as a function of the stimulus position or the stimulus type.

We conducted a 3 (stimulus position: low, middle, and high) × 2 (stimulus type: CS+, CS−) RM ANOVA to test whether SCR was modulated by CSs association (or absence thereof) with the US, as a function of its position. We observed a main effect of stimulus type (*F*_(1_,_33)_ = 44.99, *p* < 0.001, $\eta_{p}^{2}=0.58$, 90% CI [0.37, 0.69]), indicating higher SCR for CS+ than CS− (CS+: *M* = 0.60 μS, SD = 0.40 μS; CS−: *M* = 0.26 μS, SD = 0.22 μS; Figure 5).

We conducted a paired *t*-test to test whether subjective ratings of stimulus valence were modulated by CSs association (or absence thereof) with the US. Participants rated the CS+ (*M* = −2.24, SD = 2.02) as less pleasant than the CS− (*M* = 1.71, SD = 1.99; *t*_(33)_ = 6.86, *p* < 0.001, *d* = 1.17, 95% CI [0.73, 1.61]).

These results indicate that participants successfully acquired threat learning, showing higher arousal to the CS+ than the CS−, and rating the CS+ as less pleasant than CS−.

#### Subjective ratings of shock expectancy at test with electrodes

We conducted a paired *t*-test to test whether subjective ratings of shock expectancy were modulated by CSs type. Indeed, participants expected US delivery more for the CS+ (*M* = 1.97, SD = 1.91) than the CS− (*M* = −4.12, SD = 1.87; *t*_(33)_ = −14.40, *p* < 0.001, *d* = −2.47, 95% CI [−3.15, −1.78]).

#### Kinematics

**FIGURE 6:**
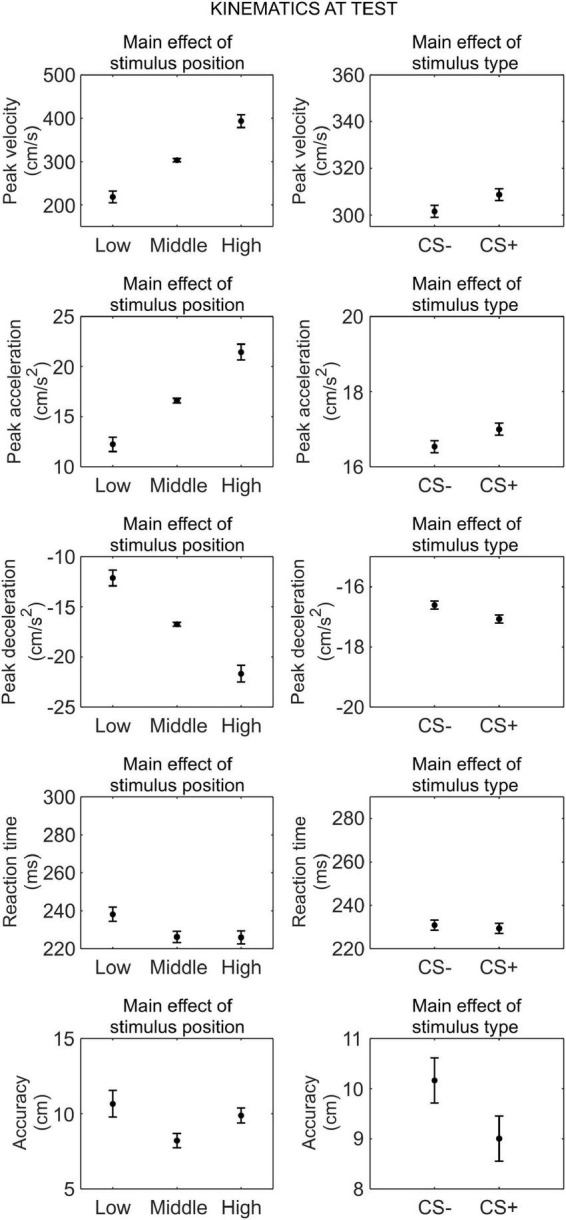
Kinematics at test. Each plot shows the group mean (black dot) and 95% confidence intervals (vertical bars) of the different kinematic parameters as a function of the stimulus position or the stimulus type. 95% CI were corrected for within-subjects designs (Cousineau, 2005).

We conducted a series of 2 (phase: test under safety, test under threat) × 3 (stimulus position: low, middle, and high) × 2 (stimulus type: CS+, CS−) RM ANOVA to assess differences in kinematics between the CS+ and CS−, after threat learning (Figure 6).

For peak velocity, we observed a main effect of position (*F*_(1_._11_,_36_._54)_ = 135.85, *p* < 0.001, $\eta_{p}^{2}=0.81$, 90% CI [0.69, 0.86]). Helmert contrasts showed that participants were slower with the low position than the middle and high positions (*t*_(66)_ = −14.14, *p* < 0.001; *M*_*low*_ = 218.45 cm/s, SD*_*low*_* = 120.06 cm/s), and with the middle position than the high position (*t*_(66)_ = −8.46, *p* < 0.001; *M*_*middle*_ = 303.54 cm/s, SD*_*middle*_* = 148.51 m/s; *M*_*high*_ = 393.37 m/s, SD*_*high*_* = 175.53 m/s). Crucially, we also observed a main effect of stimulus type (*F*_(1_,_33)_ = 7.32, *p* = 0.011, $\eta_{p}^{2}=0.18$, 90% CI [0.03, 0.36]), showing that participants were faster with the CS+ (*M* = 308.70 cm/s, SD = 146.96 cm/s) than the CS− (*M* = 301.54 cm/s, SD = 144.30 cm/s), regardless of the test phase. No other main effect or interaction was significant (all *p* ≥ 0.434).

Similarly, for peak acceleration, we observed a main effect of position (*F*_(1_._12_,_37_._06)_ = 133.74, *p* < 0.001, $\eta_{p}^{2}=0.80$, 90% CI [0.69, 0.85]). Helmert contrasts showed that participants accelerated less with the low position than the middle and high positions (*t*_(66)_ = −13.91, *p* < 0.001; *M*_*low*_ = 12.24 cm/s^2^, SD*_*low*_* = 6.71 cm/s^2^; *M*_*middle*_ = 16.61 cm/s^2^, SD*_*middle*_* = 8.14 cm/s^2^; *M*_*high*_ = 21.45 cm/s^2^, SD*_*high*_* = 9.67 cm/s^2^), and with the middle position than the high position (*t*_(66)_ = −8.59, *p* < 0.001). Crucially, we observed a main effect of CS type (*F*_(1_,_33)_ = 8.07, *p* = 0.008, $\eta_{p}^{2}=0.20$, 90% CI [0.03, 0.38]), indicating that participants accelerated more with the CS+ (*M* = 17.00 cm/s^2^, SD = 8.17 cm/s^2^) than the CS− (*M* = 16.54 cm/s^2^, SD = 7.94 cm/s^2^), regardless of the test phase. No other main effect or interaction was significant (all *p* ≥ 0.609).

For peak deceleration, we observed a main effect of position (*F*_(1_._09_,_35_._98)_ = 125.79, *p* < 0.001, $\eta_{p}^{2}=0.79$, 90% CI [0.67, 0.84]). Helmert contrasts showed that participants decelerated less with the low position than the middle and high positions (*t*_(66)_ = 13.58, *p* < 0.001; *M*_*low*_ = −12.12 cm/s^2^, SD*_*low*_* = 6.89 cm/s^2^; *M*_*middle*_ = −16.73 cm/s^2^, SD*_*middle*_* = 8.69 cm/s^2^; *M*_*high*_ = −21.67 cm/s^2^, SD*_*high*_* = 10.40 cm/s^2^), and with the middle position than the high position (*t*_(66)_ = 8.19, *p* < 0.001). Crucially, we observed a main effect of CS type (*F*_(1_,_33)_ = 10.58, *p* = 0.003, $\eta_{p}^{2}=0.24$, 90% CI [0.06, 0.42]), indicating that participants decelerated more with the CS+ (*M* = −17.07 cm/s^2^, SD = 8.74 cm/s^2^) than the CS− (*M* = −16.61 cm/s^2^, SD = 8.35 cm/s^2^), regardless of the test phase. No other main effect or interaction was significant (all *p* ≥ 0.113).

For reaction time, we observed a main effect of position (*F*_(2_,_66)_ = 10.79, *p* < 0.001, $\eta_{p}^{2}=0.25$, 90% CI [0.10, 0.37]). Helmert contrasts showed that reaction time was longer for the low position than the middle and high positions (*t*_(66)_ = 4.65, *p* < 0.001; *M*_*low*_ = 238.18 ms, SD*_*low*_* = 50.50 ms; *M*_*middle*_ = 226.21 ms, SD*_*middle*_* = 51.04 ms; *M*_*high*_ = 225.93 ms, SD*_*high*_* = 52.82 ms), while there was no difference between the middle and high positions (*t*_(66)_ = 0.09, *p* = 0.926). No other main effect or interaction was significant (all *p* ≥ 0.090).

For accuracy, we observed a main effect of position (*F*_(2_,_66)_ = 53.60, *p* < 0.001, $\eta_{p}^{2}=0.62$, 90% CI [0.48, 0.69]). Helmert contrasts showed that accuracy was higher for the low position than the middle and high positions (*t*_(66)_ = −9.48, *p* < 0.001; *M*_*low*_ = 7.16 cm, SD*_*low*_* = 9.16 cm; *M*_*middle*_ = 9.19 cm, SD*_*middle*_* = 9.71 cm; *M*_*high*_ = 10.58 cm, SD*_*high*_* = 8.94 cm), and for the middle position than the high position (*t*_(66)_ = −4.17, *p* < 0.001). No other main effect or interaction was significant (all *p* ≥ 0.348).

Thus, after threat learning, participants moved faster, accelerated more, and decelerated more when reaching the CS+ than the CS−, regardless of the test type (under threat or safety). In contrast, no difference between CS+ and CS− was found for reaction time and accuracy.

### Summary of Experiment 2

The aim of this experiment was to test whether Pavlovian threat learning increases the vigor of goal-directed actions, even when no link is established between the stimuli and action before learning. As in Experiment 1, we found a robust modulation of peak velocity, acceleration and deceleration depending on the position of the target in line with reaching in real-life (Jakobson and Goodale, 1991), confirming participants’ compliance with task instructions, and corroborating the validity of mouse tracking in studying such behavioral responses. Importantly, we replicated the results of Experiment 1 regarding the effect of threat learning on action, such that threat learning increased the peak velocity and acceleration of reaching the conditioned stimulus (CS+) relative to the control stimulus (CS−), regardless of the test phase (threatening or safe test). Additionally, we found that peak deceleration was greater for the CS+ than the CS−.

### Correlations between skin conductance response during learning and kinematics at test across Experiments 1 and 2

Given that active defensive motor responses are mediated by increased sympathetic activity (Fanselow, 1994; Rau and Fanselow, 2007; Reker et al., 2010; LeDoux and Daw, 2018; Hamm, 2020; Mobbs et al., 2020), we exploited the data collected in Experiments 1 and 2 to test whether the response of the sympathetic system during learning correlated with kinematics at test. Thus, we conduct five Pearson correlations, to assess whether the greater SCR to the CS+, relative to CS−, during threat learning correlated with the kinematics observed at test. To increase the number of data points (*n* = 68), data of both experiments were used in each correlation. Specifically, for each subject, we first calculated the difference between CS+ and CS− in mean SCR during learning and the difference between CS+ and CS− in peak velocity, acceleration, deceleration, reaction time, and accuracy at test (averaged across the three stimulus positions and the two test types). The differential SCR was then correlated with the differential kinematic parameters.

**FIGURE 7:**
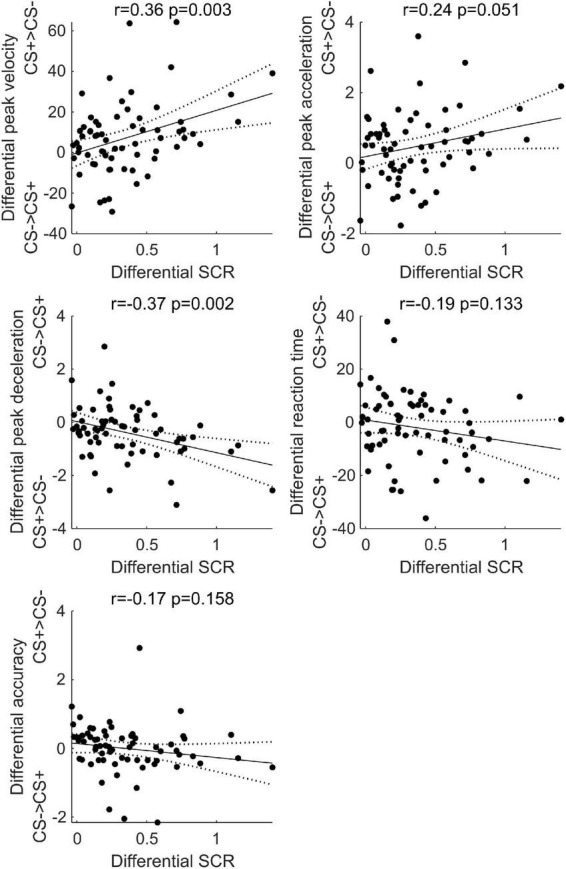
Simple linear correlation between the difference in skin conductance response between CS+ and CS– during threat learning and the difference in peak velocity, acceleration, deceleration, reaction time, and accuracy between CS+ and CS– at test. Black dots represent individual participants’ data points, the black line represents the correlation line, dashed lines represent 95% confidence intervals. Pearson’s *r* and *p*-values are reported.

As shown in Figure 7, results showed a positive correlation between differential SCR during threat learning and the differential peak velocity (*r* = 0.36, *p* = 0.003) and acceleration (*r* = 0.24, *p* = 0.051) at test, and a negative correlation with deceleration (*r* = −0.37, *p* = 0.002; two data points were removed as their residuals lied more than three standard deviations from the best-fit line OpenStax CNX, 2019). In contrast, no significant correlation was found between differential SCR during threat learning and the differential reaction time (*r* = −0.19, *p* = 0.133) and accuracy (*r* = −0.17, *p* = 0.158). Thus, the stronger was the arousal to the CS+ during threat learning, the greater was the subsequent velocity, acceleration and deceleration of reaching the CS+, relative to the CS−.

## Discussion

The aim of this study was to test whether Pavlovian threat learning shapes the kinematics of actions. In both Experiments 1 and 2, we found that threat learning increases the peak velocity and acceleration of reaching toward a conditioned stimulus. Despite this, no difference in end-point accuracy and reaction time was found following threat learning. In Experiment 2, in particular, this effect was associated with greater peak deceleration for the conditioned stimulus (this is observed also in Experiment 1, Figure 4, although the effect is not significant), explaining how movement accuracy was maintained despite the increase in velocity and acceleration. We also found that these changes in kinematics correlated with the strength of sympathetic activation during previous learning, establishing a direct relationship between past learning and subsequent changes in action. These results extend the literature on the effects of threat learning on motor responses, beyond reaction times (Krypotos et al., 2014), advancing the understanding of the mechanisms underlying adaptive and, possibly, maladaptive learning.

The increase in velocity and acceleration following threat learning may represent a response of the motor system to facilitate the implementation of active defensive responses. Indeed, in presence of a threat signaling imminent danger, defensive response mobilization appears to turn into an active action, which can result in a flight/fight response (Fanselow, 1994; Rau and Fanselow, 2007; Reker et al., 2010; LeDoux and Daw, 2018; Hamm, 2020). Our results show that motor control can be shaped by stimuli that have anticipated an impending danger in the past (i.e., shock), even when such danger is currently absent. Additionally, reaching invigoration was observed although the action had no effect on the actual occurrence of the aversive outcome, as no shock was delivered at test. Thus, the conditioned stimulus shaped reaching regardless of the actual consequences of the action (in line with Krypotos et al., 2014). This finding extends previous studies, which showed that aversively conditioned stimuli invigorate actions directed to the avoidance of the aversive outcome (Garofalo and Robbins, 2017; Gerlicher and Kindt, 2020). In particular, it shows that the invigoration of actions may emerge as a conditioned response, independently of aversive outcome avoidance. It also corroborates the idea that these changes in action are a direct consequence of learning, appearing to the mere presence of the conditioned stimulus.

We also found a positive relationship between the strength of arousal during learning and the magnitude of the subsequent kinematic changes. The greater was the arousal to the conditioned stimulus (relative to control) at learning, the greater was the velocity, acceleration and deceleration at test. This result may suggest that the increase in arousal to conditioned stimuli observed during Pavlovian learning may represent a marker of motor preparation in the face of threat. Indeed, activation of the sympathetic nervous system facilitates action and increased arousal is characteristic of active defense behaviors (Critchley, 2002). In turn, motor preparation and movements are accompanied by increases in skin conductance (Critchley, 2002). The correlation between arousal during learning and the subsequent invigoration of action also extends what is known on the relationship between emotion and action (Moors et al., 2013; Blakemore and Vuilleumier, 2017), suggesting that, in addition to current affective experience influencing action (Starita and di Pellegrino, 2018; Garofalo et al., 2019, 2020, 2021; Bertini et al., 2020; Ellena et al., 2021; Sellitto et al., 2022), also past affective experience can affect future motor responses.

Experiment 2 replicated the action invigoration effect for the conditioned stimulus, despite the absence of a baseline phase, suggesting that a stimulus-response association before Pavlovian learning is not necessary to shape action. In other words, the aversive value acquired by the conditioned stimulus may transfer onto the action, without the need of an existing representation of the stimulus in motor terms. Whether this transfer occurs during learning, test, or between the two may be the topic of future studies. Although no motor response is executed during Pavlovian learning, activity in motor and premotor cortical structures and the cerebellum has been reported (Fullana et al., 2016; Zhang et al., 2016; Fossataro et al., 2018). Additionally, structural and functional connections have been shown between the amygdala – responsible for threat learning (LaBar et al., 1998; Phelps and LeDoux, 2005) – and the cortical motor system (Bzdok et al., 2013; Diano et al., 2017), including connections with the primary motor cortex (Grèzes et al., 2014), and the supplementary motor area (Sagaspe et al., 2011). Given this evidence, we speculate that motor associations to the conditioned stimulus may be already acquired during Pavlovian learning.

In contrast to previous findings (Krypotos et al., 2014, 2015a; Karos et al., 2017; Neige et al., 2018), we found no difference in reaction time and accuracy between the conditioned and control stimuli. Given the methodological differences between our study and previous ones, including the general experimental paradigm (e.g., action directly associated with aversive outcome), type of performed action (e.g., button press and circular movement) and the tool used to perform it, identifying the cause of such difference warrants further investigation. Nevertheless, in terms of speed-accuracy tradeoff, the preserved accuracy despite the increase in velocity and acceleration appears in line with previous reports showing both a gain in accuracy, velocity and acceleration for pain-associated movements (Karos et al., 2017). Additionally, such results are in line with the evidence showing that action planning, assessed through reaction times, and execution, assessed through kinematics, are two distinct processes, that can be independently affected (e.g., Betti et al., 2018).

From a cost-benefit perspective, action invigoration (increase velocity and acceleration) in absence of any concrete danger may seem a maladaptive behavior. Indeed, more physical effort is required when the same action is made with more vigor, entailing a biological cost (Garofalo et al., in press). Nevertheless, incorrectly identifying a safe situation as dangerous is far less costly than the alternative (Dunsmoor and Paz, 2015; Laufer et al., 2016), and action invigoration may be adaptive when facilitating the implementation of active defensive responses, should the aversive outcome suddenly occur. Possibly, the action invigoration, here observed in healthy participants, may be one end of a continuum between adaptive and maladaptive behavior, as exaggerated invigoration may lead to the persistent implementation of defensive behaviors even in safe contexts. Indeed, such mechanism may resemble that of compulsive behaviors in obsessive-compulsive disorder, which are aimed at preventing some dreaded event (America Psychiatric Association, 2013), which is unlikely to actually occur. The positive correlation between the strength of arousal during learning and the magnitude of the subsequent invigoration, is in line with this idea, suggesting that highly emotional or traumatic events could lead to subsequent persistent implementation of defensive responses. Future studies may use the current paradigm in clinical populations to test whether anxiety-related disorders may indeed present an exaggerated action invigoration to conditioned stimuli.

As this is the first study on the effect of threat learning on action kinematics, we wish to comment on some limitations that may be considered by future studies, to help clarify the functional nature of action invigoration when approaching a threat, with respect to the realm of defensive behaviors (Fanselow, 1994; Krypotos et al., 2015b; Mobbs et al., 2015, 2020; Blanchard, 2017; Pittig et al., 2020). In fact, action invigoration has been previously reported also when approaching intrinsically rewarding stimuli (Reppert et al., 2015; Summerside et al., 2018; Shadmehr and Ahmed, 2020). Thus, one may wonder the extent to which our results are a consequence of the valence of the stimulus to be reached, or, rather, reflect the increase in arousal elicited by a salient stimulus. To clarify this, whether reaching invigoration can also be observed after appetitive learning should be tested. Relatedly, we did not assess SCR at test, because evaluating this measure while the participant is moving is methodologically challenging (Society for Psychophysiological Research Ad Hoc Committee on Electrodermal Measures, 2012). Nevertheless, future studies should assess whether action invigoration for the conditioned stimulus is accompanied by increased arousal, and whether the arousal response is subjected to extinction as the test progresses in the absence of shocks. Additionally, we assessed the subjective experience of participants in terms of valence ratings at the end of the learning phase in both experiments, and in Experiment 2 we also collected subjective ratings of shock expectancy relative to the test with electrodes. In the future, similar ratings could be performed before and after (or possibly during) all experimental phases, to ensure a more detailed understanding of the changes in subjective experience during the entire task. Finally, one may wonder whether the invigoration was found because participants thought that responding more quickly would lower the probability of shock. The results show that the invigoration effect resulted from a main effect of stimulus type (CS+ vs. CS−), in absence of an interaction with the test phase (under threat of shock/with shock electrodes vs. safety/without shock electrodes). Thus, it seems unlikely that an explicit belief was the driver of the invigoration, unless one assumes such belief to persist even when the shock electrodes were not attached to the participants, and thus a response-shock contingency was impossible. Nevertheless, we have not explicitly measured participants’ beliefs and are aware that inferring stimulus-driven processes (as opposed to response-driven processes) on the basis of null-effects is problematic (Buabang et al., 2022), and we thus cannot exclude such a possibility. For this reason, this issue should be addressed by future studies.

## Data availability statement

The datasets presented in this study can be found in online repositories. The names of the repository/repositories and accession number(s) can be found below: https://osf.io/av92h/.

## Ethics statement

The studies involving human participants were reviewed and approved by the Bioethics Committee of the University of Bologna. The patients/participants provided their written informed consent to participate in this study.

## Author contributions

FS, SG, and GP conceived and developed the main idea and study design. LD and DD carried out testing and data collection. FS performed the analysis under the supervision of SG and GP. FS wrote the main manuscript text in collaboration and according to the critical revisions of SG, LD, DD, and GP. All authors read and approved the final version of the manuscript.
